# Patient journey, disease burden, and functional disability in patients with axial spondyloarthritis in South Africa: results of International Map of Axial Spondyloarthritis (IMAS)

**DOI:** 10.1007/s10067-024-07151-8

**Published:** 2024-09-28

**Authors:** Kavita Makan, Marco Garrido-Cumbrera, Riette Du Toit, José Correa-Fernández, Maranda van Dam, Mohammed Tikly

**Affiliations:** 1 Division of Rheumatology, Department of Internal Medicine, Faculty of Health Sciences, University of the Witwatersrand, Johannesburg, South Africa; 2 Health & Territory Research (HTR), University of Seville, Seville, Spain; 3 Axial Spondyloarthritis International Federation (ASIF), London, UK; 4 Division Rheumatology, Department Medicine, Faculty of Medicine and Health Sciences, Stellenbosch University, Parow, South Africa; 5 Axial Spondyloarthritis Association of South Africa, Grootbrakrivier, South Africa

**Keywords:** African, Diagnostic delay, Health-related quality of life, Spondyloarthritis

## Abstract

**Objective:**

To assess the unmet needs of South Africans with axial spondyloarthritis (axSpA) focusing on the patient journey, functional disability, and health-related quality of life.

**Methods:**

One hundred forty-six South African axSpA patients completed the International Map of Axial Spondyloarthritis (IMAS) online survey. Patient journey, functional disability, activities of daily living, and psychological stress were analyzed in relation to socio-demographic characteristics, disease activity, diagnostic delay, extra-musculoskeletal manifestations, and drug therapy.

**Results:**

Majority were female (82.2%) and Caucasian (89.7%) and the mean age of participants, age at onset of initial symptoms, and diagnostic delay were 44.7, 26.7, and 10.8 years, respectively. Participants reported a mean of 3.4 visits to healthcare professionals prior to a definitive diagnosis of axSpA, mostly made by rheumatologists (77.9%). Active disease (BASDAI ≥ 4) was reported by 87%, 69.9% suffered from psychological distress (general health questionnaire-12 score ≥ 3), and more than two-thirds suffered functional limitations in daily, personal, and social activities. Multivariable logistic analysis showed that active disease was more common in females [OR (95% CI) = 4.3 (1.2–15.2)] and was associated with greater functional limitation [OR (95% CI) = 1.1 (1.0–1.2)].

**Conclusion:**

Of all the regions assessed in the IMAS (*n* = 5557 participants, 27 countries), South Africans reported the longest delay in diagnosis. The South African patient journey depicts a process burdened with diagnostic challenges and delays, coupled with patients experiencing significant personal and social limitations. These results emphasize the urgent need to establish local diagnostic and treatment guidelines for axSpA in South Africa, to reduce diagnostic delay, and to control disease activity associated with functional limitation in axSpA.

**Table:** 

**Key Points** • *Axial spondyloarthritis (axSpA) in South Africans is associated with significant limitations in physical, mental, and social functioning.* • *First study to describe the unmet needs of South African patients with axSpA.*

**Supplementary Information:**

The online version contains supplementary material available at 10.1007/s10067-024-07151-8.

## Introduction

Axial spondyloarthritis (axSpA) is a chronic inflammatory disease predominantly affecting the axial skeleton and sacroiliac joints [1]. It is further characterized by the presence of extra-musculoskeletal manifestations such as anterior uveitis, psoriasis, and inflammatory bowel disease [2]. Symptoms usually appear between 20 and 30 years of age, with a mean diagnostic delay of around 7 years [3, 4].

Not much is known about the prevalence, clinical spectrum, or disease burden of axSpA in sub-Saharan Africa. Small cross-sectional and retrospective studies suggest that axSpA is uncommon, partly due to the rarity of HLA-B27 in this region, the genetic risk factor strongly associated with axSpA in Caucasians. The relative absence of HLA-B27 and limited access to MRI could also lead to underdiagnosis and underrepresentation of the true burden of the disease [5]. Notably, South Africa has the largest Caucasian population among African countries (4.5 million) [6]. Considering global figures and using a conservative estimate of 1 per 500 inhabitants, this would equate to a total of 160,000 axSpA patients in South Africa [7]. Moreover, a recent, albeit small study showed a higher incidence of peripheral arthritis, enthesitis, and uveitis in axSpA patients in South Africa among the regions surveyed [8].

The impact of axSpA on health-related quality of life (HR-QoL) can be profound [9] and is related to both disease activity [10] and delay in diagnosis [11]. Until recently, only nonsteroidal anti-inflammatory drugs (NSAIDs) were consistently shown to improve the symptoms of pain and stiffness in axSpA. However, the introduction of targeted biologic therapies in patients with axSpA, with inadequate response to NSAIDs, has been associated with dramatic improvements in patient HR-QoL [12].

The healthcare system in South Africa is disproportionately fragmented, with most of the population supported by the state-funded public sector, while the private sector, largely financed by individual contributions to healthcare or health insurance schemes, covers approximately 16% of South Africans [13]. Regardless of the sector, access to biologic therapies is challenging. In the state sector, access to biologics is limited to a very small minority of axSpA patients treated at tertiary hospitals because of cost. By contrast, in the private healthcare sector, biologics are restricted to patients with top comprehensive medical insurance coverage. Unlike rheumatoid arthritis, axSpA is not listed in the 26 chronic conditions governed by the Prescribed Minimum Benefits (PMB) rules of the Medical Schemes Act of South Africa. Medical schemes in South Africa are compelled under this Act to provide chronic funding for diagnosis and treatment of these conditions, regardless of the type of medical insurance plan [14].

Given the dearth of data on the prevalence and impact of axSpA on HR-QoL in South Africa and limited access to biologics, the aim of the present study was to understand the patient journey of South Africans with axSpA including time to diagnosis, access to biological treatment, functional disability, mental health, and quality of life in order to identify current unmet needs.

## Methods

### Study design and survey development

This international project was initiated in Spain with the Atlas of axSpA 2017 pilot study [15]. Subsequently, 12 European countries joined the project becoming the European Map of Axial Spondyloarthritis (EMAS) [16]. Due to the wide dissemination of EMAS, countries from all over the world joined the project, resulting in a final sample of 5557 participants with axSpA from 27 countries, known as the International Map of Axial Spondyloarthritis (IMAS), of whom 146 participants were from South Africa. IMAS is a joint effort between the University of Seville’s Health & Territory Research group, Axial Spondyloarthritis International Federation (ASIF), and a steering committee made up of axSpA experts from across the world and patient representatives.

### Setting

IMAS collected information using an online cross-sectional survey. Participants completed a comprehensive questionnaire containing over 120 items on socio-demographics, health behaviors, diagnosis and disease characteristics, comorbidities, mental health (General Health Questionnaire, GHQ-12), healthcare utilization, pharmacological treatments, disease activity (Bath Ankylosing Spondylitis Disease Activity Index, BASDAI), physical activity and functioning, employment, and disease-related fears and hopes. More information on the items, their domains, and the specific questions can be found in the seminal EMAS article and IMAS article [16, 17].

### Participants and recruitment in South Africa

**Fig. 1:**
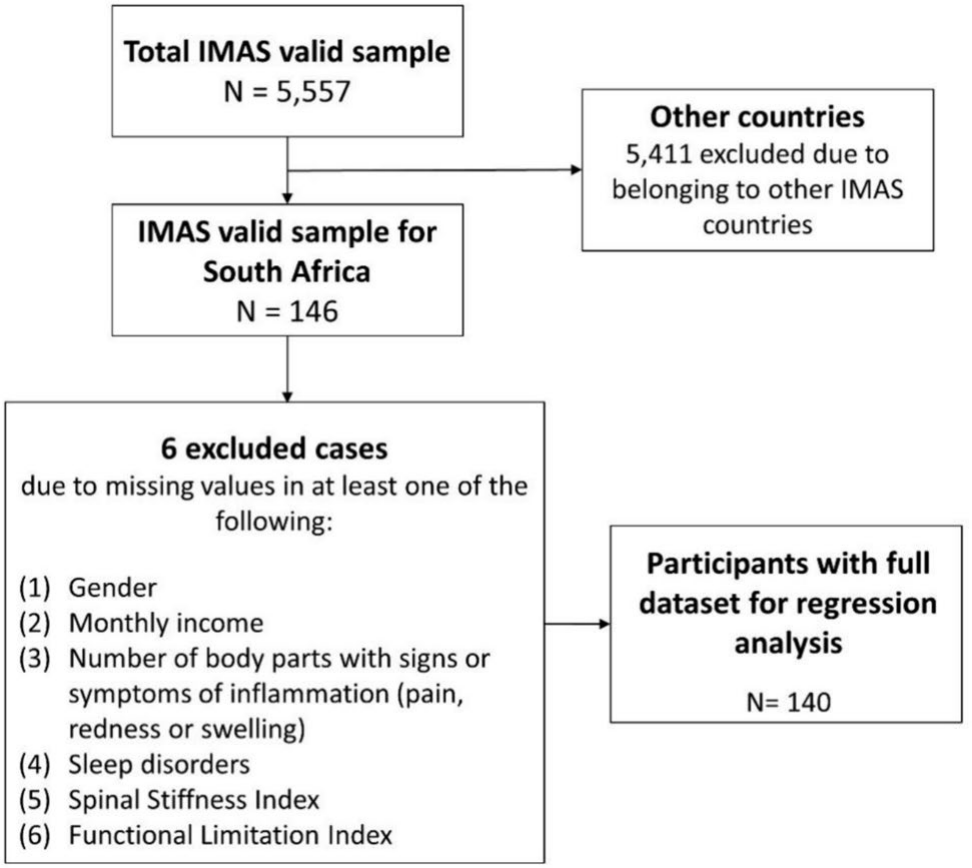
South Africa IMAS participant recruitment flowchart

The survey was conducted in South Africa between October 1 and December 31, 2021. Participants were recruited by the Axial Spondyloarthritis Association of South Africa (ASASA), a patient organization, via an online survey prepared by Ipsos, a market research company. Persons 18 years and older, residing in South Africa and with a self-reported diagnosis of axSpA (either AS or nr-axSpA) were invited to complete the survey (see Fig. 1).

### Variables

In the present study, sociodemographic variables, physical health, disease extra-musculoskeletal manifestations, patient journey, functional disability, quality of life, patient-reported outcomes, mental health, and medication are reported. The measurement and categories of variables are shown in Table S1 of the supplementary material.

The following patient-reported outcomes were documented:

- Spinal Stiffness Index: an index developed by the University of Seville specifically for the IMAS survey to assess the degree of spinal stiffness experienced by patients in the spinal column, distinguishing between the cervical, dorsal, and lumbar areas. For these three areas, the degree of stiffness was requested: no stiffness (scored as 1), mild (scored as 2), moderate (scored as 3), and severe (scored as 4). The index is obtained as the sum of the scores collected in the three areas, with a range between 3 and 12 points. Higher values of the index indicate greater spinal stiffness [15].
- Functional Limitation Index: an index developed by the University of Seville specifically for the IMAS survey to assess the degree of limitation in 18 activities of daily life. For these 18 areas, the functional limitation was requested: no restriction (scored as 0), low (scored as 1), medium (scored as 2), and high (scored as 4). The index is obtained as the sum of the scores obtained in the 18 areas, with a range between 0 and 54 points. Higher values of the index indicate higher functional limitation [15].
- Bath Ankylosing Spondylitis Disease Activity Index (BASDAI): a self-administered questionnaire that evaluates disease activity in patients with axSpA. It includes six questions relating to the following symptoms: fatigue; pain in the spinal column; inflammation/pain in joints other than the neck, back, and hips; areas of localized tenderness (also called enthesitis, or inflammation of tendons and ligaments); and the level and duration of stiffness in the morning, all assessed on a 0–10 numeric rating scale [18]. The overall BASDAI has a range from 0 to 10. Cut-off point at 4 indicates active disease (BASDAI ≥ 4).
- 12-item General Health Questionnaire (GHQ-12): a screening measure of common mental health disorders in the general population, including symptoms of anxiety, depression, social dysfunction, and loss of confidence [19, 20]. Overall GHQ-12 has a range from 0 to 12. Cut-off point at 3 indicates the risk of poor mental health (GHQ score ≥ 3).

### Statistical analysis

Descriptive statistics are shown as frequencies (percentages) for qualitative variables and as mean (SD) for quantitative variables. Bivariate analyses were conducted to determine possible relationships between the study variables and disease activity (BASDAI cut-off). For quantitative variables (age, monthly income, body mass index, number of body parts with signs or symptoms of inflammation (pain, redness, or swelling), spinal stiffness, functional limitation, and diagnostic delay), the Mann–Whitney test was used to evaluate differences in the distribution of variables between BASDAI cut-off points (< 4 and ≥ 4). For categorical variables (gender, educational level, physical activity, uveitis, inflammatory bowel disease, psoriasis, GHQ-12 cut-off, depression, anxiety, sleep disorders, NSAIDs, disease-modifying antirheumatic drugs—DMARDs and Biologics), chi-square test was applied to compare the distribution of variables between GHQ-12 cut-off points. Variables that showed statistical significance (*p* < 0.05) in the bivariate analysis were introduced in the binary logistic regression model: gender (female), monthly income, number of body parts with signs or symptoms of inflammation (pain, redness, or swelling), sleep disorders (yes), spinal stiffness (3–12), and Functional Limitation Index (0–54). Each variable was introduced individually into the model to determine which was associated with active disease (BASDAI ≥ 4). Data were analyzed using SPSS software V.26, and differences were considered statistically significant at the 5% level.

## Results

**Fig. 2:**
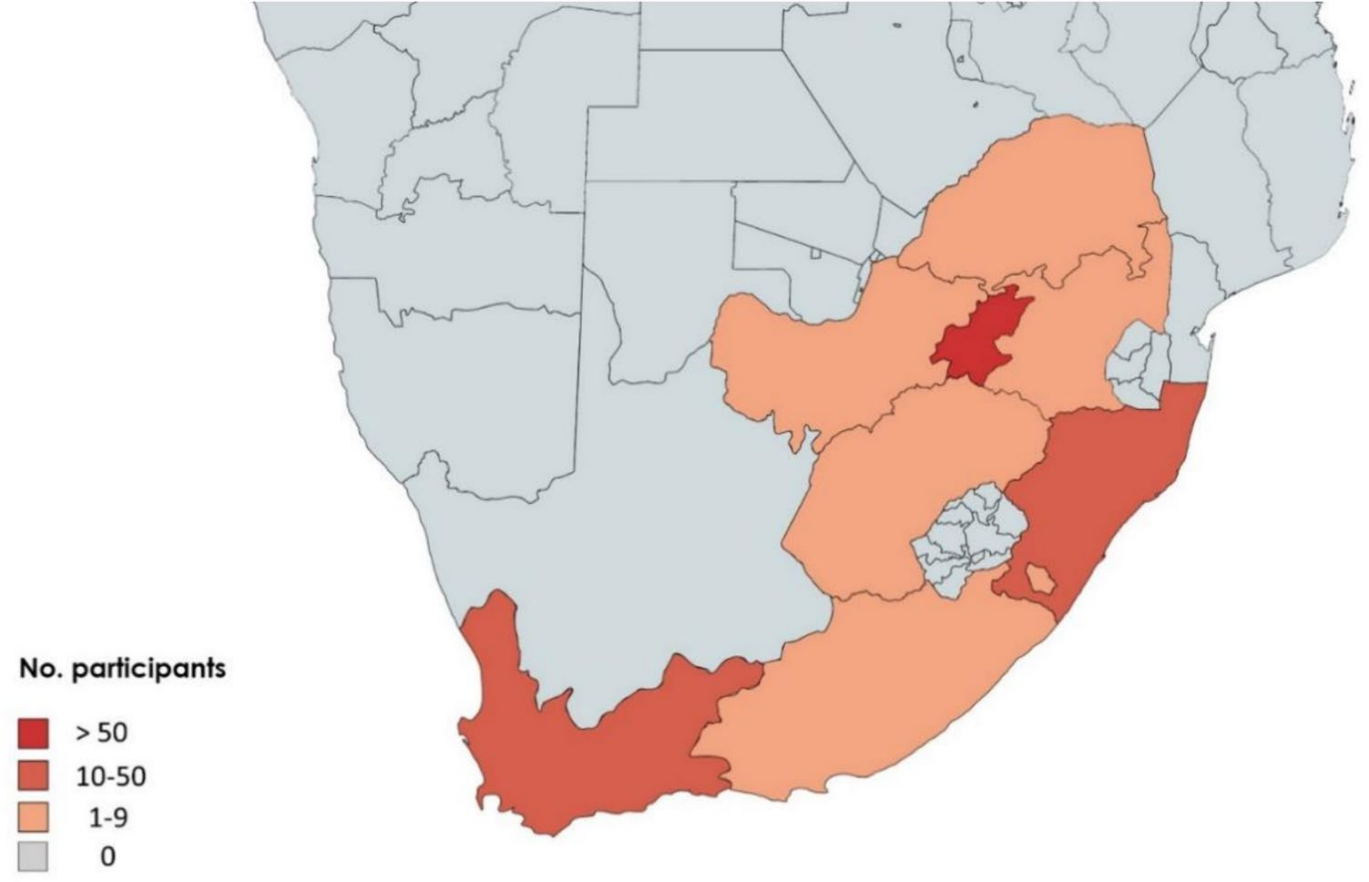
Distribution and sociodemographic characteristics of participants in South Africa (*n* = 146)

Responses were received from 8 of 9 regions that divide South Africa: Gauteng, Western Cape, KZN, Free State, Northwest, Mpumalanga, Eastern Cape, and Limpopo; the majority of participants resided in Gauteng, the Western Cape, and KwaZulu Natal (KZN) (Fig. 2).

Mean (SD) age of participants was 44.7 (10.3) years, and most were Caucasian, female, married, and employed. Approximately, one in three had graduate qualifications and earned a monthly income of more than 2600 USD (R50,000.00 South African Rands). With respect to disease characteristics, 72.2% of participants had a positive HLA-B27 test, 25.2% had a history of uveitis, 24.1% had inflammatory bowel disease, and 11.9% had psoriasis. Furthermore, the majority (87%) of participants had active disease (BASDAI ≥ 4) and most reported previous treatment with NSAIDs (94.4%) and DMARDs (79.3%), while fewer had received biologics (42.5%) for disease management (Table 1).

**Table 1:** Socio-demographic, disease characteristics, and treatment of axSpA participants in South Africa (*n* = 146, unless specified otherwise)

	n (%)
**Socio-demographics**	
Ethnicity: Caucasian	131 (89.7)
Age: mean (SD)	44.7 (10.3)
Gender: female	120 (82.2)
Marital status: married	97/143 (67.8)
Educational level: graduates	54 (37.0)
Employment status: employed	81/120 (67.5)
Monthly income (> 2600 USD)	45 (30.8)
**Disease characteristics**	
HLA-B27 positive	83/135 (72.2)
Uveitis	34/135 (25.2)
Inflammatory bowel disease	33/137 (24.1)
Psoriasis	16 (11.9)
BASDAI (≥ 4)	127 (87.0)
**Drug therapy**	
NSAIDs	136/144 (94.4)
DMARDs	111/140 (79.3)
Biologics	62 (42.5)

*NSAIDs* nonsteroid anti-inflammatory drugs, *DMARDs* disease-modifying antirheumatic drugs

**Fig. 3:**
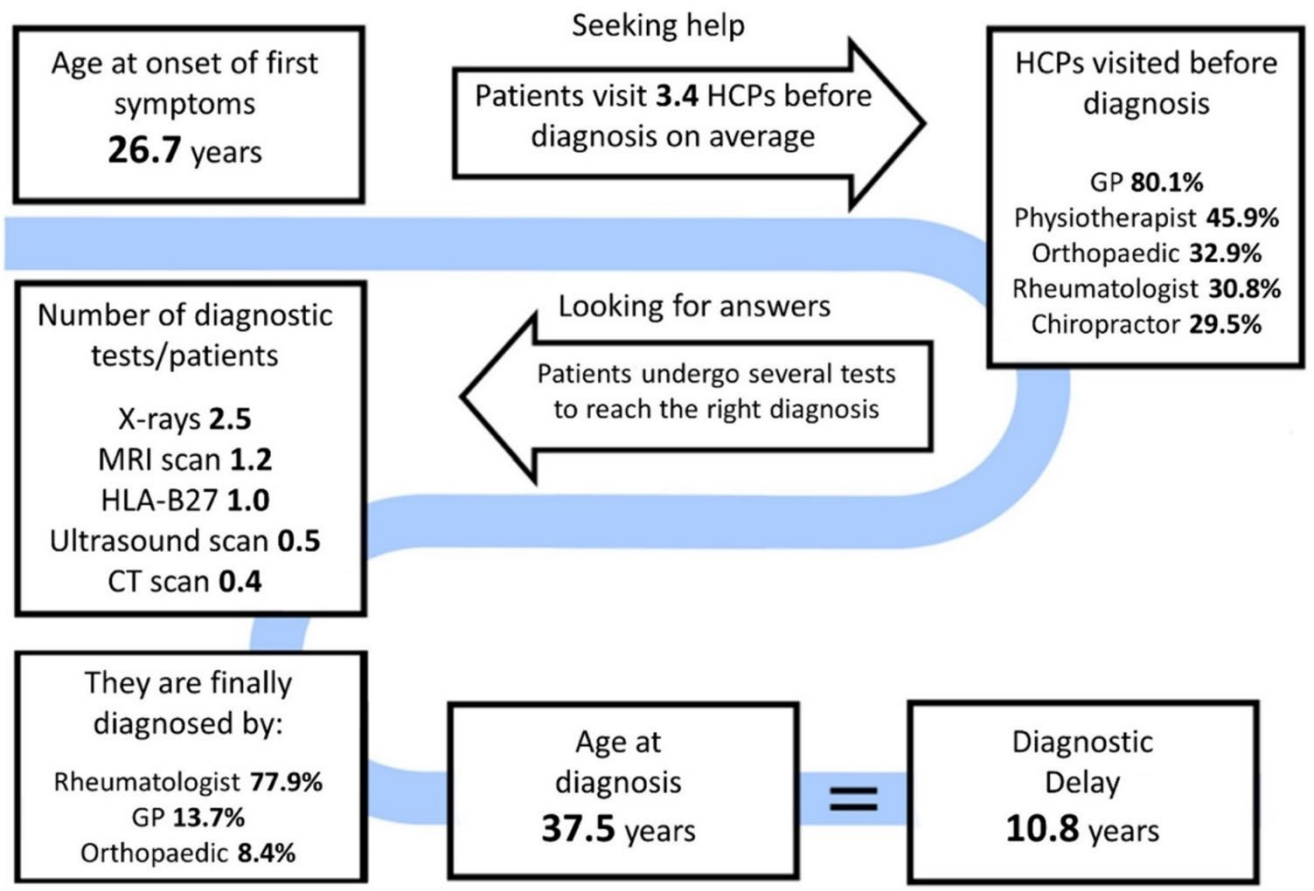
Patient journey for axial spondyloarthritis in South Africa

South African IMAS participants reported a mean (SD) of 26.7 (11.4) years for the onset of first axSpA symptoms and 3.4 (2.2) visits to healthcare professionals (HCPs) before the diagnosis of axSpA was made. Mean (SD) age at diagnosis was 37.5 (10.8) years, equating to a mean diagnostic delay of 10.8 (10.6) years, (10.9 for females vs 9.9 for males, *p* = 0.5, respectively). Prior to diagnosis, 117 (80%) of participants reported previous consultations with general practitioners (GPs), 67 (45.9%) with physiotherapists, 48 (32.9%) with orthopedic surgeons, 45 (30.8%) with rheumatologists, and 43 (29.5%) with chiropractors. Prior to axSpA diagnosis, the mean (SD), [% of participants who had test] number of x-rays per participant was 2.5 (3.5), [38.3%], 1.2 (1.7), [34.8%] for MRI scans and 1.0 (0.7), [82.6%] for HLA-B27. Definitive diagnosis of axSpA was made by rheumatologists (*n* = 102, 77.9%), followed by GPs (*n* = 18, 13.7%) and orthopedic surgeons (*n* = 11, 8.4%) (Fig. 3).

As shown in Table 2, most participants experienced limitations in daily activities, often affecting physical exercise and housework or cleaning. Participants reported feeling guilty about the impact of the disease on family and friends, with many avoiding social engagements (45.9%) or staying out for a long period of time (57.5%), and approximately 72% were unable to participate in activities/hobbies once previously enjoyed.

**Table 2:** Functional limitations in daily activities and personal and social impact of axSpA as reported by 146 participants in South Africa

	Yes, *n* (%)
The top five most common functional limitations in daily activities (high or moderate limitations)	
Doing physical exercise	100 (68.5)
Housework/cleaning	87 (59.6)
Going up or down the stairs	71 (48.6)
Shopping	65 (44.5)
Engaging in intimate relations	61 (41.8)
The five aspects of personal life most often affected by SpA/AS	
I feel guilty about the impact of AS on my family/close friends/partner	110 (75.3)
I avoid making commitments	88 (60.3)
It has affected my sex life	87 (59.6)
I feel like my family/close friends/partner do not understand my situation	76 (52.1)
It’s made me distant to my family/close friends/partner	65 (44.5)
The five social activities most often affected by SpA/AS	
I cannot take part in all activities/hobbies that I used to	104 (71.2)
When I do go out, I do not stay out for very long	84 (57.5)
It has stopped me from going to social events	67 (45.9)
I do not plan any events/outings in advance	63 (43.2)
I don’t go out/see friends as much	50 (34.2)

Those with active disease (BASDAI ≥ 4) were more frequently female (*p* = 0.003) with lower monthly incomes per household (*p* = 0.049) and reported (1) a higher number of body parts with signs or symptoms of inflammation (*p* = 0.002), (2) presence of sleep disorders (*p* = 0.01), (3) a higher degree of spinal stiffness (*p* = 0.001) and (4) greater functional limitation (*p* < 0.001) (Table 3). In the univariable logistic analysis, factors associated with active disease were female gender (OR = 4.4, 95% CI = 1.6, 12.4), higher number of body parts with signs or symptoms of inflammation (OR = 1.2, 95% CI = 1.1, 1.3), presence of sleep disorders (OR = 4.2, 95% CI = 1.3, 13.4), greater spinal stiffness (OR = 1.5, 95% CI = 1.2, 2.0), and greater functional limitation (OR = 1.1, 95% CI = 1.1, 1.2). In the multivariable logistic analysis, female gender (OR = 4.3, 95% CI = 1.2, 15.1) and greater functional limitation (OR = 1.1, 95% CI = 1.01, 1.2) were associated with active disease (Table 4).

**Table 3:** Bivariate analysis to evaluate possible relationships with active disease (BASDAI ≥ 4), *n* = 146 unless specified otherwise

	BASDAI < 4	BASDAI ≥ 4	*P* value
**Socio-demographic characteristics**			
Age, mean (SD) in years	47.4 (11.8)	44.3 (10.1)	0.4
Diagnostic delay, mean (SD) in years	12.6 (12.5)	10.5 (10.4)	0.5
Ethnicity: Caucasian	19 (100.0)	112 (88.2)	0.4
Gender Male Female	8 (42.1) 11 (57.9)	18 (14.2) 109 (85.8)	**0.003**
Educational level No schooling completed Primary school High school University	0 (0.0) 0 (0.0) 14 (73.7) 5 (26.3)	0 (0.0) 0 (0.0) 78 (61.4) 49 (38.6)	0.3
Monthly income per household $, *n* = 121, mean (SD)	3402.0 (1747.9)	2496.7 (1728.0)	**0.049**
**Physical health**			
Body mass index kg/m^2^, mean (SD)	26.7 (5.6)	29.9 (9.5)	0.2
Physical activity, *n* = 145	16 (84.2)	99 (78.6)	0.6
No. of body parts with signs/symptoms of inflammation (pain, redness, or swelling), mean (SD)	7.6 (5.9)	11.8 (4.9)	**0.002**
Extra-musculoskeletal manifestations			
Uveitis, *n* = 135	3 (15.8)	31 (26.7)	0.3
Inflammatory bowel disease, *n* = 137	3 (15.8)	30 (25.4)	0.4
Psoriasis, *n* = 135	2 (10.5)	14 (12.1)	0.8
Mental health			
GHQ-12 ≥ 3	10 (52.6)	92 (72.4)	0.08
Anxiety, *n* = 140	6 (33.3)	68 (55.7)	0.08
Depression, *n* = 142	9 (47.4)	67 (54.5)	0.6
Sleep disorders, *n* = 140	4 (21.1)	64 (52.9)	**0.01**
**Patient-reported outcomes**			
Spinal stiffness, mean (SD)	7.3 (1.8)	8.8 (1.7)	**0.001**
Functional limitation, mean (SD)	7.4 (8.3)	20.9 (12.8)	** < 0.001**
**Medical therapy, ever**			
NSAIDs, *n* = 144	18 (94.7)	118 (94.4)	1.0
DMARDs, *n* = 140	17 (89.5)	94 (77.7)	0.2
Biologics	11 (57.9)	51 (40.2)	0.1

*NSAIDs* nonsteroidal anti-inflammatory drugs, *DMARDs* disease-modifying antirheumatic drugs, *GHQ-12* 12-item General Health Questionnaire

**Table 4:** Regression analysis for variables associated with active disease (*n* = 140)

	Univariable logistic analysis		Multivariable logistic analysis	
	OR	95% CI	OR	95% CI
Gender: female	4.404	1.559, 12.439	4.269	1.202, 15.162
Monthly income per household	1.000	0.999, 1.000	-	-
No. of body parts with signs or symptoms of inflammation (pain, redness, or swelling)	1.191	1.066, 1.330	1.077	0.946, 1.226
Sleep disorder present	4.211	1.321, 13.421	2.904	0.763, 11.054
Spinal stiffness	1.549	1.174, 2.041	1.255	0.910, 1.731
Functional limitation	1.135	1.060, 1.215	1.088	1.012, 1.170

## Discussion

This survey of South Africans with axSpA highlights several unmet physical and psychological needs related to the disease. Most participants (87%) reported active disease, with over two-thirds having functional limitations in daily physical, personal, and social activities and more than two-thirds reporting poor mental health (GHQ score ≥ 3). This is an interesting observation as most participants were from a relatively affluent background and over 40% had reported the use of biologics. Moreover, the mean delay in diagnosis from symptom onset was more than a decade, with most participants visiting several HCPs prior to a definitive diagnosis of axSpA.

Studies from several countries have reported mean diagnostic delays ranging from 6 to 8 years, being longer in women than in men. Several factors may potentially explain the substantially longer diagnostic delay (> 10 years) and higher number of visits to HCPs before axSpA diagnosis (> 3 visits) in South Africa compared to other countries. Firstly, compared to other countries, axSpA is generally uncommon and underrecognized in South Africa, with a lower index of suspicion among HCPs, except for rheumatologists. In the current study, the diagnosis of axSpA was made by a rheumatologist in more than 75% of participants. This is a worrying statistic with the knowledge that only 115 trained rheumatologists are registered in a country of 56 million inhabitants [21]. Secondly, HLA-B27 testing is less helpful in diagnosing axSpA, particularly nr-axSpA, given its low prevalence (< 1%) in black South Africans, in whom HLA-B27 is not a genetic risk factor for axSpA [5]. That almost three-quarters tested positive for HLA-B27 in the present study is because close to 90% of participants were Caucasian. Thirdly, access to MRI for detecting axSpA is limited due to the high cost associated with the equipment, especially in the state sector, as well as restrictions in the private sector by private medical insurance in funding MRI scans, rendering it unaffordable to many patients. This is confirmed by our findings in the present study, where just over a third of patients had MRI scan to confirm the diagnosis of axSpA.

Additional factors that might be barriers to early diagnosis and appropriate management of axSpA in South Africa include socio-economic inequalities, a high cost of private medical insurance, and scarcity of rheumatological expertise, especially outside of the larger metropolitan areas [22].

Functional limitations that were explored in South African IMAS participants demonstrated substantial limitations in activities of daily living, such as engaging in physical activity, cleaning, or merely using the stairs. Participants also reported feeling guilty about the impact axSpA had on their relationships with friends and relatives, with many refraining from making commitments, participating in hobbies, or going out of the house for long periods of time. Data from the larger European IMAS cohort showed that high levels of disease activity impacted negatively on social life, especially in participants who were female, divorced/separated, unemployed/on sick leave, and those with poor mental health and/or anxiety disorders [23]. Multivariable regression analysis in the present study showed that active disease was associated with female gender and greater functional limitation. Additionally, univariable regression analyses showed that active disease was independently associated with disordered sleep, spinal stiffness, and a greater number of body parts with signs or symptoms of inflammation. Some studies have shown that disease activity is associated with obesity [24], cardiovascular disease [25], accelerated radiographic spinal progression [26], and mental disorders such as depression [27].

Of serious concern is that most participants (82.3%) who had received biologic therapy in the present survey reported ongoing active disease, a much lower response than reported in studies from industrialized Western countries [28]. Likely reasons for this are the introduction of biologics late in the course of the disease, intermittent access to drugs because of cost and variable funding by Medical Aid schemes, and restricted access to non-TNF biologics [14].

Physical exercise seems to be an effective non-pharmacological therapy for axSpA [29] and therefore, high-intensity physical exercise may improve disease activity and reduce the risk of cardiovascular disease [30]. Consequently, HCPs such as rheumatologists, physiotherapists, and/or primary care physicians may see benefit in recommending a structured exercise program—including a combination of flexibility, muscle strength, and aerobic exercises to improve spine mobility, reduce disease activity, and enhance functional capacity [31, 32].

There are several limitations of this South African IMAS study. Firstly, most respondents were Caucasian South Africans who represent less than 10% of the total population. This bias is because the members of ASASA are mainly Caucasian, who, in general, are financially better off compared to other ethnic groups and have greater access to private medical insurance, specialized care, and advanced imaging such as MRI [33]. Secondly, the disproportionately high number of female respondents can be explained by the disease being increasingly diagnosed in women [34], and as previous studies have suggested, women tend to be more likely to respond to online surveys [35]. Thirdly, patients from lower socio-economic groups tend to have less access to the internet and social media [36], further exaggerating the sample bias in favor of the more affluent Caucasian South Africans. Fourthly, online surveys have been shown to provide a lower response rate compared to in-person surveys where the interviewer has the opportunity to review responses or resolve respondents’ doubts [37, 38]. Fifthly, the survey was based on self-reported diagnosis and was not intended to confirm diagnosis or to validate responses with clinical assessments. Finally, the sample size of the South African IMAS survey was relatively small, partly as a result of the limited time of only 3 months for data collection, which was shorter than most other countries that participated in the IMAS survey.

Despite these limitations, these data, generated through the IMAS project, are the first to emphasize the personal experience and opinions of axSpA patients in South Africa, as well as to elucidate the factors associated with active disease. Given that most of the respondents were mostly affluent Caucasians, it is likely that delay in diagnosis and management of axSpA is worse in indigent South Africans who have limited access to specialized care. Hence, a larger study of axSpA patients analyzing and comparing those in the public and private healthcare sectors is needed to understand the unmet needs of the average patient in South Africa.

## Conclusion

South Africa has the longest diagnostic delay of all regions assessed in the International Map of Axial Spondyloarthritis, with a mean of 10.8 years waited before diagnosis. This unacceptable diagnostic delay is coupled with a challenging patient journey to diagnosis and worrisome functional limitations in daily, personal, and social life. In addition, active disease in these patients is associated with female gender and high functional limitation. Increased availability of MRI and specific training of GPs would enhance early identification of suspected axSpA cases, although complicated access to biologic therapy in diagnosed patients is a barrier to effective management in South African patients. It is essential to establish guidelines to improve the diagnosis of patients, as well as to monitor disease activity, which is associated with functional limitation, particularly in South African females.

## Supplementary Information

Below is the link to the electronic supplementary material.

Supplementary file1 (DOCX 34 KB)

## Data Availability

All source data files have been provided in the manuscript and supplementary material.
