# Supplementary material for: Patient journey, disease burden, and functional disability in patients with axial spondyloarthritis in South Africa: results of International Map of Axial Spondyloarthritis (IMAS)
Source: Clin Rheumatol. 2024 Sep 28;43(11):3335–43. doi: 10.1007/s10067-024-07151-8 (PMC11489170; doi:10.1007/s10067-024-07151-8)
Supplement: Supplementary file 1 — Supplementary file1 (DOCX 34 KB) [file 10067_2024_7151_MOESM1_ESM.docx]

**Patient Journey, Disease Burden and Functional Disability in Patients with Axial Spondyloarthritis in South Africa. Results of International Map of Axial Spondyloarthritis**

Table S1: Measurement and categories of reported variables

| **Variables** | **Questions** | **Categories/measures** |
| --- | --- | --- |
| **Socio-demographic** |  |  |
| Region | In which region do you live? | Mpumalanga, Limpopo, Gauteng  Northwest, Eastern cape, Western cape, Free state, KZN, Northern cape |
| Age | Please specify your age | In years |
| Gender | Please specify your gender | Male, Female |
| Ethnicity | Please specify your ethnicity | White, Black, Indian, Asian, Mixed |
| Education level | Please select your level of education completed | No schooling, Primary school, High school, University |
| Marital status | Please select your marital status | Single, Married, Separated/divorced, Widowed |
| Employment status | What is your current employment status? (Please specify main situation) | Employed, Employed - full time, Employed - part time, Self-employed, Temporary sick leave, Permanent sick leave, Retired, Early retirement, Unemployed, Homemaker, Student, Other |
| Income level | What is the total monthly net income of your household? | No income, R 8,000 or less, R 8,001 – 16,500, R 16,501 – 25,000, R 25,001 – 33,500, R 33,501 – 50,000, R 50,001 – 84,000, R 84,001 or over, No reply |
| **Physical health** |  |  |
| Physical activity | Do you do any physical or sporting activity, including walking? | Yes, no |
| Body mass index | Please specify your height.  Please specify your weight. | Centimetre  Kilogram |
| No. of body parts with inflammation of pain | In which part of your body have you noticed redness, swelling or pain (inflammation) at some point due to Spondylitis/ spondyloarthritis? | Pelvis, spine, Cervical (Neck), Dorsal region (upper back), Thoracic spine (middle of the back), Lumbar region (lower back), Sacroiliac joints (pain in buttocks), Hips, Thorax (chest), Ribs, Uveitis/Iritis (eyes), Mandible (lower jaw), Metacarpals (joints in hands), Phalanges (fingers), Inflammation / pain in toe joints, Vertebrae, Shoulders, Wrists, Ankles, Knee joints, Dorsum (back or top) of foot, Achilles tendon, Sole / heel of foot, Elbows, Tendons, Others |
| **Disease extra-musculoskeletal manifestations** |  |  |
| Uveitis | Please indicate whether you have been diagnosed with any of the following: | Uveitis |
| Inflammatory bowel disease | Please indicate whether you have been diagnosed with any of the following: | Inflammatory bowel disease |
| Psoriasis | Please indicate whether you have been diagnosed with any of the following: | Psoriasis |
| **Patient journey** |  |  |
| HCPs before diagnosis | Which of the following health professionals did you see for your Spondylitis/ Spondyloarthritis before it was diagnosed? | Primary care physician / family doctor / GP, Rheumatology nurse, Orthopaedic specialist, Physiotherapist at a GP practice/Community Physiotherapist, Private Physiotherapist, Physical and Rehabilitation Medicine (PRM) specialist, Internal medicine specialist, Neurosurgery specialists, Osteopath, Chiropractor, Nurse Practitioner/ Physician Assistant, Obstetrician-gynaecologist, Ophthalmologist, Pain specialist, Gastroenterologist, Dermatologists  Sports Therapist / Masseur |
| Number of tests to diagnosis | What were the tests done to diagnose your Spondylitis/ spondyloarthritis? | Magnetic resonance (MRI scan), Radiographic test (X-ray), Genetic analysis (HLA B27), Ultrasound scan, Radionuclide scintigraphy, Computed tomography (CT scan), Other |
| Diagnostic parameters | What was the result of the genetic test (HLA-B27)? | Positive, Negative, do not know |
| HCP who diagnosed the disease | Which medical professional made the diagnosis of Spondylitis/ spondyloarthritis | Primary care physician / family doctor /GP, Rheumatologist, Orthopaedic specialist, Physiotherapist, Nurse Practitioner/ Physician Assistant, Do not know, Other |
| Age at onset of first symptoms | Age of onset of first symptoms (pain, inflammation, stiffness) associated with spondylitis/ spondyloarthritis | In years |
| Age at diagnosis | Age at which you were diagnosed with spondylitis/ spondyloarthritis | In years |
| Diagnostic delay | Calculated based on the age at diagnosis | In years |
| **Functional limitations** |  |  |
| Functional limitations in daily activities | Please indicate whether you are restricted by your Spondylitis/ spondyloarthritis? (No restriction, low, medium, and high) | Dressing/undressing, Washing/personal grooming, Taking a bath/shower, Tying shoelaces, Walking/getting around the house, Going up or down the stairs, Lying down/getting up from bed, Going to the toilet, Shopping, Cooking, Eating, Housework/cleaning, Walking down the street, Using public transportation, Driving, Doing physical exercise, Engaging in intimate relations, Going to the doctor, Playing or caring for your children/grandchildren |
| Functional limitations personal life | In what ways has Spondylitis/ spondyloarthritis axial SpA (AS) impacted your personal life? Please select all that apply. | It’s made me closer to my family/close friends/partner, It’s made me distant to my family/close friends/partner, It has affected my sex life, I avoid making commitments, I find it hard to date/find a life partner, I feel guilty about the impact of Spondylitis/ spondyloarthritis, I feel like my family/close friends/partner do not understand my situation, I feel like my family/close friends/partner are very understanding, I do not have a close circle of family/close friends/partner, Other |
| Functional social life | In what ways has Spondylitis/ spondyloarthritis  axial SpA (AS) impacted your social life? Please select all that apply. | I cannot take part in all activities/hobbies that I used to, It has stopped me from going on holiday, It has stopped me from going to social events, I do not plan any events/outings in advance, I do not invite friends to my house, When I do go out, I do not stay out for very long, I don’t go out / see friends as much, My Spondylitis/ spondyloarthritis does not hold me back |
| **Medication** |  |  |
| NSAIDs | Have you ever been treated with a Non-Steroidal Anti-Inflammatory Drug (NSAID) for your spondylitis/ spondyloarthritis? | Yes, no |
| Biologics | Have you ever been treated with a Biologic for your spondylitis/ spondyloarthritis? | Yes, no |
| DMARDs | Have you ever been treated with a Conventional Synthetic Disease Modifying Anti-Rheumatic Drug (DMARD) for your spondylitis/ spondyloarthritis? | Yes, no |
| **Mental comorbidities** |  |  |
| Anxiety | Please indicate whether you have been diagnosed with any of the following: | Anxiety |
| Depression | Please indicate whether you have been diagnosed with any of the following: | Depression |
| Sleep disorders | Please indicate whether you have been diagnosed with any of the following: | Sleep disorders |

R: South African Rand (ZAR); NSAID: Non-Steroidal Anti-Inflammatory Drug; DMARD: Disease Modifying Anti-Rheumatic Drug; AS: axial SpA; MRI: Magnetic resonance imaging; PRM: Physical and Rehabilitation Medicine.
